# Global publication trends and research hotspots of the gut-liver axis in NAFLD: A bibliometric analysis

**DOI:** 10.3389/fendo.2023.1121540

**Published:** 2023-03-09

**Authors:** Shuangjie Yang, Deshuai Yu, Junjie Liu, Yanfang Qiao, Shuxiao Gu, Ran Yang, Xinlou Chai, Wei Wang

**Affiliations:** ^1^ School of Traditional Chinese Medicine, Beijing University of Chinese Medicine, Beijing, China; ^2^ Dongzhimen Hospital, Beijing University of Chinese Medicine, Beijing, China; ^3^ Department of Cardiology, Nanjing Pukou Hospital of Traditional Chinese Medicine, Nanjing, China; ^4^ Guang’anmen Hospital, China Academy of Chinese Medical Sciences, Beijing, China

**Keywords:** NAFLD, the gut-liver axis, bibliometric analysis, visualization, citespace, VOSviewer

## Abstract

**Background:**

Nonalcoholic Fatty Liver Disease(NAFLD)refers to a spectrum of diseases ranging from simple liver steatosis to nonalcoholic steatohepatitis (NASH) and cirrhosis. Bidirectional cross-talk between the gut-liver axis plays an important role in the pathogenesis of NAFLD. To learn more about the gut-liver axis in NAFLD, this study aims to provide a comprehensive analysis from a bibliometric perspective.

**Method:**

Literature related to the gut-liver axis in NAFLD from 1989 to 2022 was extracted from the Web of Science Core Collection. Based on Microsoft Excel, CiteSpace and Vosviewer, we conducted to analyze the number of publications, countries/regions, institutions, authors, journals, references, and keywords.

**Results:**

A total of 1,891 literature since 2004 was included, with the rapid growth of the number of papers on the gut-liver axis in NAFLD annually. These publications were mainly from 66 countries and 442 institutions. Of the 638 authors analyzed, Bernd Schnabl was the one with the most publications, and Patrice D. Cani was the one with the most co-citations. *International Journal of Molecular Sciences* is the journal with the most articles published, and *Hepatology* is the journal with the most citations. The most common keywords are gut microbiota, inflammation, and insulin instance, which are current research hotspots. Short-chain fatty acid, *in vitro*, randomized controlled trial in clinical, and diabetes mellitus represent the research frontiers in this field and are in a stage of rapid development.

**Conclusion:**

This is the first study to conduct a comprehensive bibliometric analysis of publications related to the gut-liver axis in NAFLD. This study reveals that gut microbiota, inflammation, insulin resistance, short-chain fatty acids, and randomized controlled trial will be the hotspots and new trends in the gut-liver axis in NAFLD research, which could provide researchers with key research information in this field and is helpful for further exploration of new research directions.

## 1 Introduction

Nonalcoholic Fatty Liver Disease (NAFLD) is a common chronic metabolic disease. According to statistics (1), its prevalence in adults around the world is about 25%. As of the end of 2020, a total of 1 billion people in the world are affected by it, causing great impact on social medical care and economy, it has become a worldwide public issue. NAFLD is a multi-systemic metabolic disease (2), its pathogenesis involves multiple factors such as obesity, insulin resistance, inflammation, etc. At present, the mechanism of NAFLD is not clear yet, and there is no effective treatment for this disease currently exists (3). Therefore, further exploration on the pathogenesis of NAFLD plays an important role in its prevention and treatment.

Gut-liver axis describes the crosstalk relationship among the liver, the gut, and the gut microbiota (4). It plays a vital role in the onset and progress of NAFLD (5). Clinical studies in the past ten years have found that the changes in intestinal microbial flora of NAFLD patients are mainly manifested as the decrease of bacterial diversity (6). At the same time, studies have shown that high fat diet (HFD) causes microbial group disorders in NAFLD mice, leading to damage of intestinal epithelial barrier and gut vascular barrier (GVB) damage, which promotes inflow of pathogen-associated molecular patterns (PAMPs), further exacerbated inflammation responses (7). Farnesoid X receptor (FXR) is the primary bile acid receptor in the liver and small intestine. Studies have shown that inhibition of the FXR signal conduction in the intestine could reduce the synthesis of liver fatty acids and the ceramide in the intestine, thereby reducing the accumulation of liver lipids and improving HFD induced NAFLD (8). In addition, many studies suggest that the intestinal flora and its metabolites play an important role in the onset and progress of NAFLD, which are key targets in the NAFLD treatment (5).

Bibliometrics is a discipline that conducts qualitative and quantitative analysis on the literature system and characteristics through mathematics and statistics (9). It can reflect the development of a certain field in a global context through visual analysis of the countries, institutions, journals, and authors, etc., it could also access and predict the foundation and emerging trend of scientific research through the co-occurrence and emergence analysis on the references and keywords (10). VOSviewer and CiteSpace are two most commonly used bibliometric analysis software (11). Through the bibliometric analysis, researchers can quickly learn about the development status of disciplines and use it to guide future researches. This study is based on the visual analysis with CiteSpace and VOSviewer software to clarify the research situation and trend of NAFLD and gut liver axis over the past 20 years, thus to provide new ideas for the development of NAFLD drugs.

## 2 Materials and methods

### 2.1 Data extraction

Literature was extracted from the Web of Science Core Collection database and was downloaded on October 24, 2022. The search strategies was as follows: TS= (NASH) OR TS= (non-alcoholic steatohepatitis) OR TS= (nonalcoholic steatohepatitis) OR TS= (NAFLD) OR TS= (non-alcoholic fatty liver disease) OR TS=(nonalcoholic fatty liver disease)OR TS=(MAFLD) OR TS=(metabolic associated fatty liver disease))AND ((TS= Liver (Topic) or TS= (hepatic*)) AND (TS= (gastro* micro*) or TS= (gastro* flora*) or TS=(gut micro*) or TS=(gut flora) or TS=(intestin * micro*) or TS=(intestin * flora))) OR (TS=(gut−liver axis) or TS=(gut liver axis)).We totally retrieved 2037 records. Then we eliminated invalid documents, including meeting abstracts (88), editorial materials (38), corrections (6), and letters (14). By this filter, 1891 records were included, of which articles (1209) accounted for 63.93% of the total, followed by reviews (682, 36.06%). This data extraction and exclusion were independently by two authors (SJY and DSY) and any disagreement was resolved by consulting the corresponding author (JJL). All data was exported and stored in the Data folder in the download_txt format at last.

### 2.2 Data analysis

This study conducted visual analysis on data with Microsoft Excel 2021, VOSviewer1.6.18 and CiteSpace6.1. R3.

**Figure 1:**
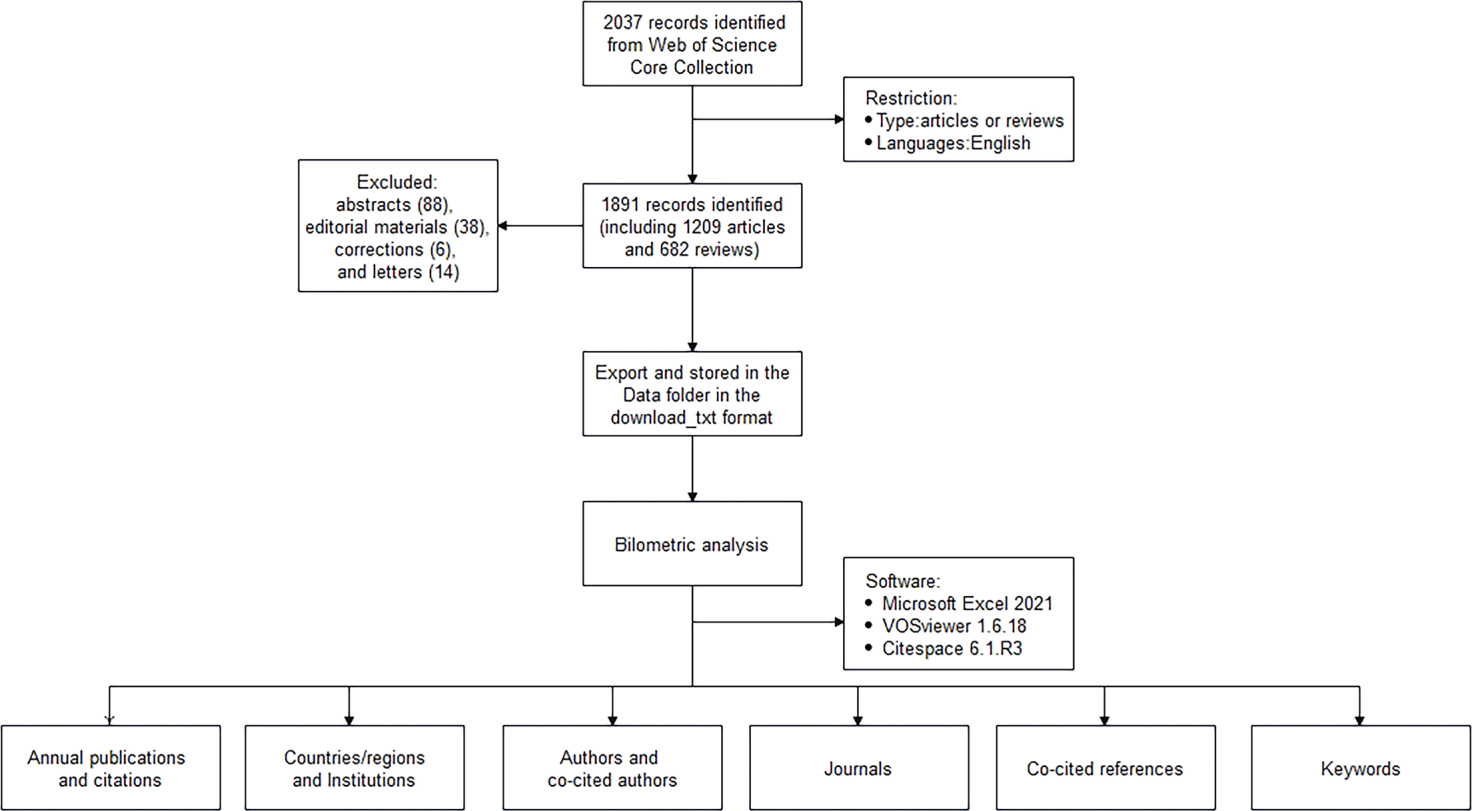
Flow chart for the analysis of the gut-liver axis in NAFLD researches.

We analyzed the number of annual publications, the H indexes of countries and authors, the number of co-citations, etc. In all retrieved articles, we found one with publication time of 2023, it was incorporated into 2022 analysis to facilitate data process. VOSviewer and CiteSpace was used to realize the visual analysis of distribution of the countries/regions, authors and co-cited authors, institutions, journals and co-cited journals, co-cited references, and keywords. For the keywords and co-cited references, we then performed clustering and burst detection. The clustering labels refer to the three algorithms provided in the Citespace software, including log-likelihood ratio (LLR), latent semantic indexing (LSI), and mutual information (MI) (12). **Figure 1** shows the flow chart of literature screening and data analysis process.

## 3 Results

### 3.1 Annual publication growth and citation analysis

**Figure 2:**
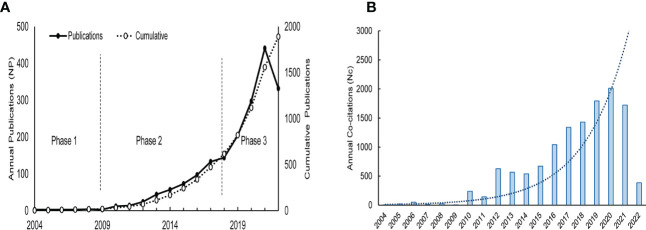
Articles related to the study of the gut-liver axis in NAFLD. **(A)** The Annual and cumulative publication numbers from 2004 to 2022. **(B)** Annual number of citations (Nc).

A total of 1,891 articles were included. The total number of non-self-cited citations of retrieved articles was 36,726, and the average times cited per article is 34.22. The H-index of all articles is 115. **Figures 2A, B** show the annual number of publications (Np) and annual citations (Nc) of the gut-liver axis in NAFLD related research articles. As shown in **Figure 2**, the first research article in this field was published in 2004, and its development was divided into three stages with 2009 and 2018 as the boundaries. The first stage is the infant stage from 2004 to 2009, the number of articles in this stage was relatively small, and studies on the gut-liver axis in the field of NAFLD just started. The second stage is the development stage from 2010 to 2018, the annual growth rate of the number of publications increased steadily. The third stage is the outbreak stage from 2019 to 2022, the number of publications on the gut-liver axis in the field of NAFLD increased significantly, and the total number of annual publications reached 441 in 2021. It shows that more and more researchers have begun to pay attention to the potential of the gut-liver axis in the field of NAFLD.

### 3.2 Distribution of countries/regions

**Figure 3:**
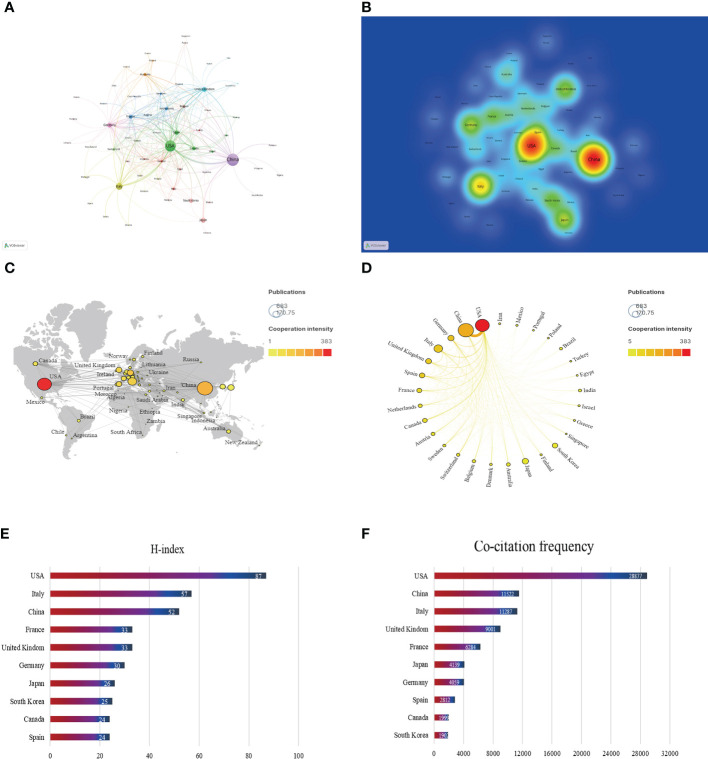
Contribution of different countries to the study of the gut-liver axis in NAFLD. **(A)** The network of collaboration map of countries/regions based on VOSviewer. **(B)** The density visualization of all participating countries. **(C)** Geographical distribution map of global publications related to the gut-liver axis in NAFLD. **(D)** The top 30 countries with the most publications. **(E)** Top 10 countries for H index. **(F)** Top 10 in terms of co-citation frequency.

In the past 20 years, a total of 66 countries/regions have published articles on research of the gut-liver axis in NAFLD, as shown in **Figures 3A, B** and **Table 1**, China is the country with the largest number of publications (674/35.64%), followed by the United States (520/27.50%) and Italy (187/9.9%). In terms of total citations, the United States publications were cited 28,877 times, followed by China (11,522) and Italy (11,287). **Figures 3C, D** shows that Europe, the United States, and Asia are the main countries and regions of publications. The color of the nodes and the thickness of lines represent the strength of cooperation between countries, indicating that the United States and China have relatively closer cooperation. The H-index is a new method for evaluating the academic achievements of researchers, so we also conducted statistical analysis on the H-index, as shown in **Figures 3E, F**, the H-index of the United States ranks first (87), followed by Italy (57) and China (52). Combining these indicators, we can see that the United States is a relatively leading country in this research field.

**Table 1:** Top 10 countries with the most published research on the gut-liver axis in NAFLD.

Rank	Country	Np	Nc	Average citation	h-index	Total link strength
1	China	674	11522	17.09	52	205
2	USA	520	28877	55.53	87	383
3	Italy	187	11287	60.36	57	132
4	Japan	109	4139	37.97	26	31
5	Germany	100	4059	40.59	30	154
6	United Kindom	93	9001	96.78	33	123
7	South Korea	84	1902	22.64	25	20
8	Spain	81	2812	34.72	24	121
9	France	67	6284	93.79	33	91
10	Canada	63	1999	31.73	24	77

### 3.3 Authors and co-cited authors

#### 3.3.1 Authors

**Figure 4:**
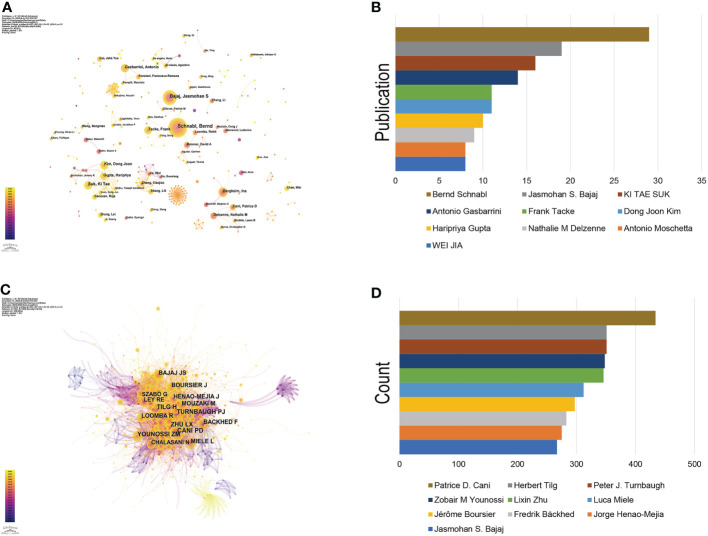
Authors involved in the study of the gut-liver axis in NAFLD. **(A)** Visualization of co-occurrence of authors based on CiteSpace. **(B)** Top 10 authors in terms of number of publications. **(C)** A visual map of co-cited authors based on CiteSpace. **(D)** Top 10 authors in terms of co-citations.

Since the first article published in the field of the gut-liver axis in NAFLD in 2004, 638 authors have conducted studies related to the gut-liver axis in NAFLD. Visual analysis on the authors was conducted *via* CiteSpace, each node represents an author, and the larger the node, the higher the number of publications. The map (**Figure 4A**) had 638 nodes and 1,299 edges, and the network density was 0.064. As shown in **Figure 4B** and **Table 2**, Bernd Schnabl (29 publications) had the largest number of publications, followed by Jasmohan S. Bajaj (19 publications), Ki Tae Suk (16 publications), and Antonio Gasbarrini (14 publications). The links between nodes represent the collaboration between the authors, indicating that there is collaborative relationship between the authors. The thicker the line, the closer the cooperation. The top 2 collaborative teams are Bernd Schnabl’s team from the University of California San Diego and KI TAE SUK’s team from Hallym University. Most of the authors included in this study are presented as scattered nodes, suggesting that the collaboration among authors needs to be further strengthened.

**Table 2:** Top 10 authors with the most published research on the gut-liver axis in NAFLD.

Rank	Author	Institutions	Publication
1	Bernd Schnabl	University of California San Diego	29
2	Jasmohan S. Bajaj	Virginia Commonwealth University	19
3	KI TAE SUK	Hallym University	16
4	Antonio Gasbarrini	Università Cattolica del Sacro Cuore	14
5	Frank Tacke	Charité – Universitätsmedizin Berlin	11
6	Dong Joon Kim	Hallym University	11
7	Haripriya Gupta	Hallym University	10
8	Nathalie M Delzenne	Université catholique de Louvain	9
9	Antonio Moschetta	Università degli Studi di Bari Aldo Moro	8
10	WEI JIA	Shanghai Jiao Tong University	8

#### 3.3.2 Co-cited authors

Co-cited author refers to two or more authors who are cited by at least one article at the same time, indicating that there are similarities in the research of these two authors. **Figure 4C** shows network of co-cited authors visually through CiteSpace. The top 10 co-cited authors were cited more than 200 times (**Figure 4D**, **Table 3**). Patrice D. Cani ranks first in the top 10 co-citations (434), followed by Peter J. Turnbaugh (351) and Herbert Tilg (351). The highest centrality was achieved by Shi Qi Yang (0.85), Ernst J. Drenick (0.81) and Keary Cope (0.69), indicating that these authors played a role of bridge in the field.

**Table 3:** Top 10 co-cited authors on the gut-liver axis in NAFLD.

Rank	Author	Institutions	Count
1	Patrice D. Cani	Université Catholique de Louvain	434
2	Peter J. Turnbaugh	University of California San Francisco	351
3	Herbert Tilg	Innsbruck Medical University	351
4	Zobair M Younossi	Falls Church	348
5	Lixin Zhu	Tongji University	346
6	Luca Miele	Università Cattolica del Sacro Cuore	312
7	Jérôme Boursier	University of Angers	297
8	Fredrik Bäckhed	University of Gothenburg	283
9	Jorge Henao-Mejia	University of Pennsylvania	275
10	Jasmohan S. Bajaj	Virginia Commonwealth University	267

### 3.4 Active institutions

**Figure 5:**
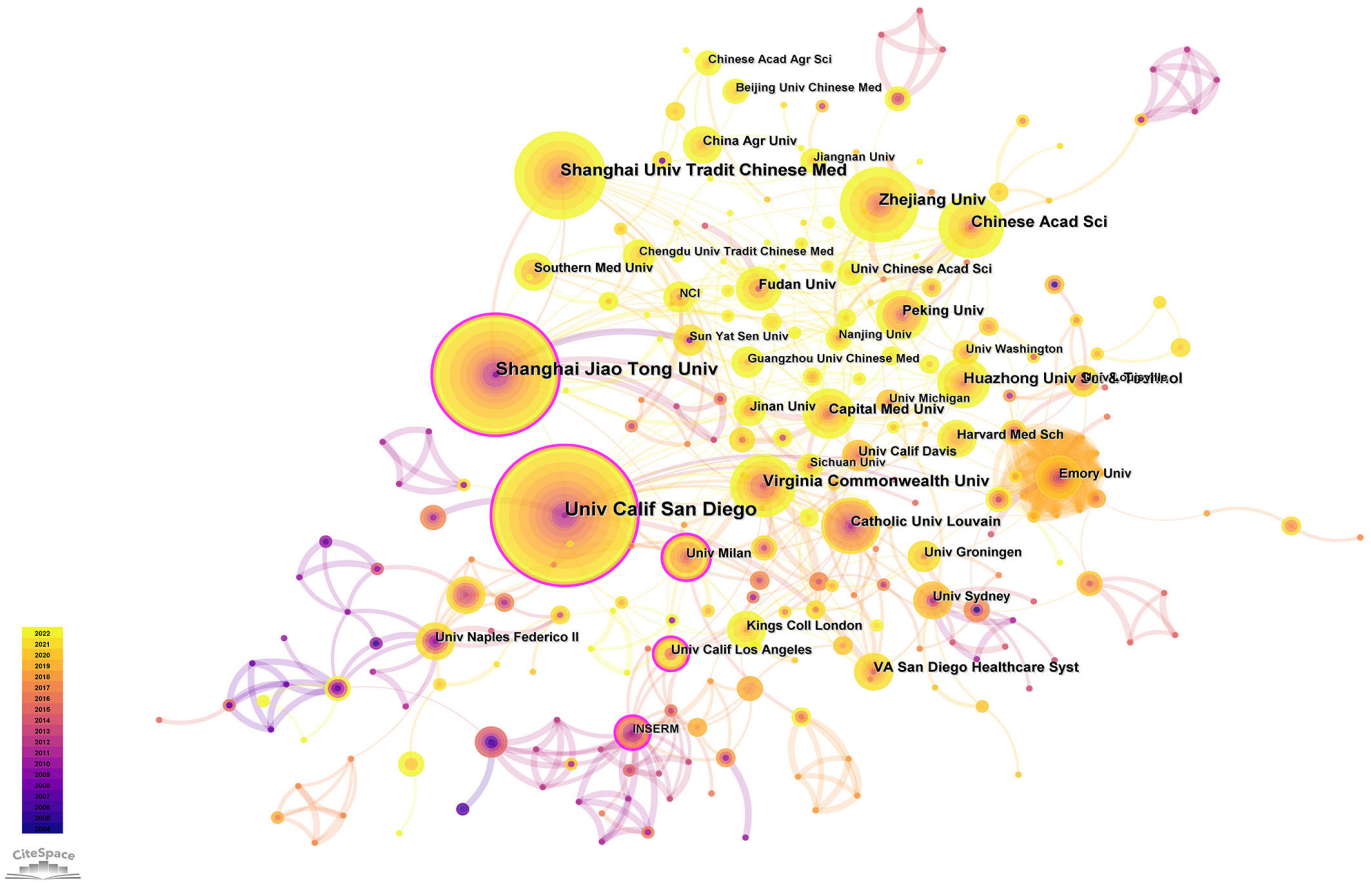
The network of institutions conducting research related to the gut-liver axis in NAFLD.

Institutional co-occurrence network analysis was conducted by CiteSpace (**Figure 5**) to find organizations or institutions with relatively mature research. **Table 4** lists the top 10 institutions in terms of number of publications and centrality of the gut-liver axis in NAFLD research. The nodes in the figure represent institutions, and larger nodes represent more publications of the institution; the links between nodes represent cooperation among institutions, the color of links indicates the start time of the collaboration, and the thickness of lines indicates the strength of the collaboration. The University of California San Diego (60 publications) takes lead in number of publications, followed by Shanghai Jiao Tong University (50 publications) and Shanghai University of Traditional Chinese Medicine (39 publications).

**Table 4:** Top 10 institutions conducting the gut-liver axis in NAFLD by volume and centrality.

Rank	Institutions	Count	Rank	Institutions	Centrality
1	University of California San Diego	60	1	California State University, Los Angeles	0.51
2	Shanghai Jiao Tong University	50	2	University of California San Diego	0.39
3	Shanghai University of Traditional Chinese Medicine	39	3	Catholic University of Louvain	0.38
4	Zhejiang University	31	4	Cleveland Clinic	0.38
5	Chinese Academy of Sciences	30	5	VA San Diego Healthcare System	0.37
6	Virginia Commonwealth University	29	6	Hartford Hospital	0.36
7	Huazhong University of Science and Technology	24	7	University of California, Davis	0.32
8	Hallym University	21	8	DENT Neurologic Institute	0.32
9	Catholic University of Louvain	19	9	Institut national de la santé et de la recherche médicale	0.31
10	Fudan University	19	10	Institut National de la Recherche Agronomique	0.29

The purple circle outside the node indicates a high degree of centrality (≥0.10), which may lead to transformative discoveries and may act as a bridge. The top three institutions for centrality are California State University, Los Angeles (0.51), University of California San Diego (0.39) and Catholic University of Louvain (0.38). As in the figure, the cooperation between institutions is relatively close.

### 3.5 Journals

**Figure 6:**
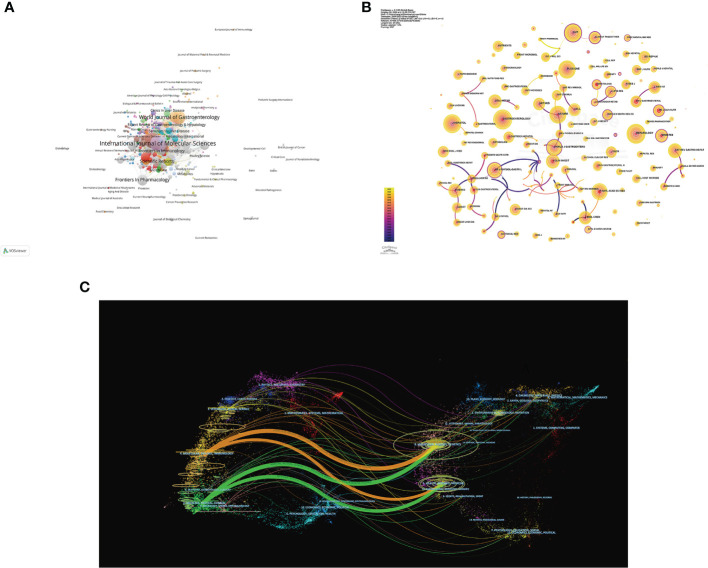
Visualization of journals of the gut-liver axis in NAFLD. **(A)** Network visualization analysis of source journals based on VOSviewer. **(B)** Visualization of cited journals based on CiteSpace. **(C)** The dual-map overlay of journals in the gut-liver axis and NAFLD.

Source analysis of the included literature showed that *International Journal of Molecular Sciences* (77 publications) was the journal with the largest number of publications in this field, followed by *Nutrients* (65 publications) and *World Journal of Gastroenterology* (47 publications) (**Figure 6A**). The JCR partitions of the top 10 journals with the largest number of publications are all Q1 or Q2, which indicates that the quality of the publications included in this study is reliable, and it also suggests that researchers can give priority to such journals when publishing articles.

In **Figure 6B**, co-citation analysis of journals showed that *Hepatology* (1,575 total citations) was the most cited journal, followed by *Gastroenterology* (1,430 total citations) and *Journal of Hepatology* (1,357 total citations). Among top 10 journals with the most citations, *Nature Reviews Gastroenterology & Hepatology* has the highest IF of 73.082. 70% of top 10 journals with the most citations were classified as Q1, and the remaining 3 journals were classified as Q2 (**Table 5**). The journal with the highest centrality is *Cell Death & Differentiation* (0.97), followed by *Archives of Disease in Childhood-Fetal and Neonatal Edition* (0.94) and *Archives of Biochemistry and Biophysics* (0.8) (**Table 5**), which also shows these journals have higher influence in the field.

**Table 5:** Top 10 journals for co-citation of the gut-liver axis in NAFLD.

Rank	Journal	Count	JCR Partitions	Rank	Journal	Centrality	JCR Partitions
1	Hepatology	1040	Q1	1	Cell Death & Differentiation	0.97	Q1
2	Gastroenterology	1034	Q1	2	Archives of Disease in Childhood-Fetal and Neonatal Edition	0.94	Q1
3	Journal of Hepatology	1018	Q1	3	Archives of Biochemistry and Biophysics	0.8	Q2
4	Plos ONE	993	Q2	4	American Journal of Physiology-Cell Physiology	0.77	Q1
5	Gut	974	Q1	5	BMJ-British Medical Journal	0.73	Q1
6	Nature	870	Q1	6	Alimentary Pharmacology & Therapeutics	0.53	Q1
7	PNAS	861	Q1	7	Biochemical and Biophysical Research Communications	0.4	Q3
8	World Journal of Gastroenterology	742	Q2	8	Biostatistics	0.4	Q1
9	Nature Reviews Gastroenterology & Hepatology	738	Q1	9	Cancer Research	0.39	Q1
10	Scientific Reports	724	Q2	10	Gut	0.37	Q1

The Dual-map of CiteSpace could reflect the development of the research in different disciplines. As shown in **Figure 6C**, citing articles are shown on the left, cited articles are shown on the right, and the colored curved path in the middle indicates the citation relationship. The four orange or green citation paths indicate that research in Molecular, Biology, Genetics journals and Health, Nursing, Medicine journals are frequently cited by Molecular/Biology/Immunology journals and Medicine/Medical/Clinical journals. At the same time, disciplines such as Veterinary/Animal/Science, Ecology/Earth/Marine, Physics/Materials/Chemistry, Environmental/Toxicology/Nutrition and Psychology/Education/Social displayed in the edge regions of the overlay plots are also involved in the research of the gut-liver axis in NAFLD, which to a certain extent shows that researchers have carried out multidisciplinary cross-cooperative studies in this field.

### 3.6 Co-cited references

**Figure 7:**
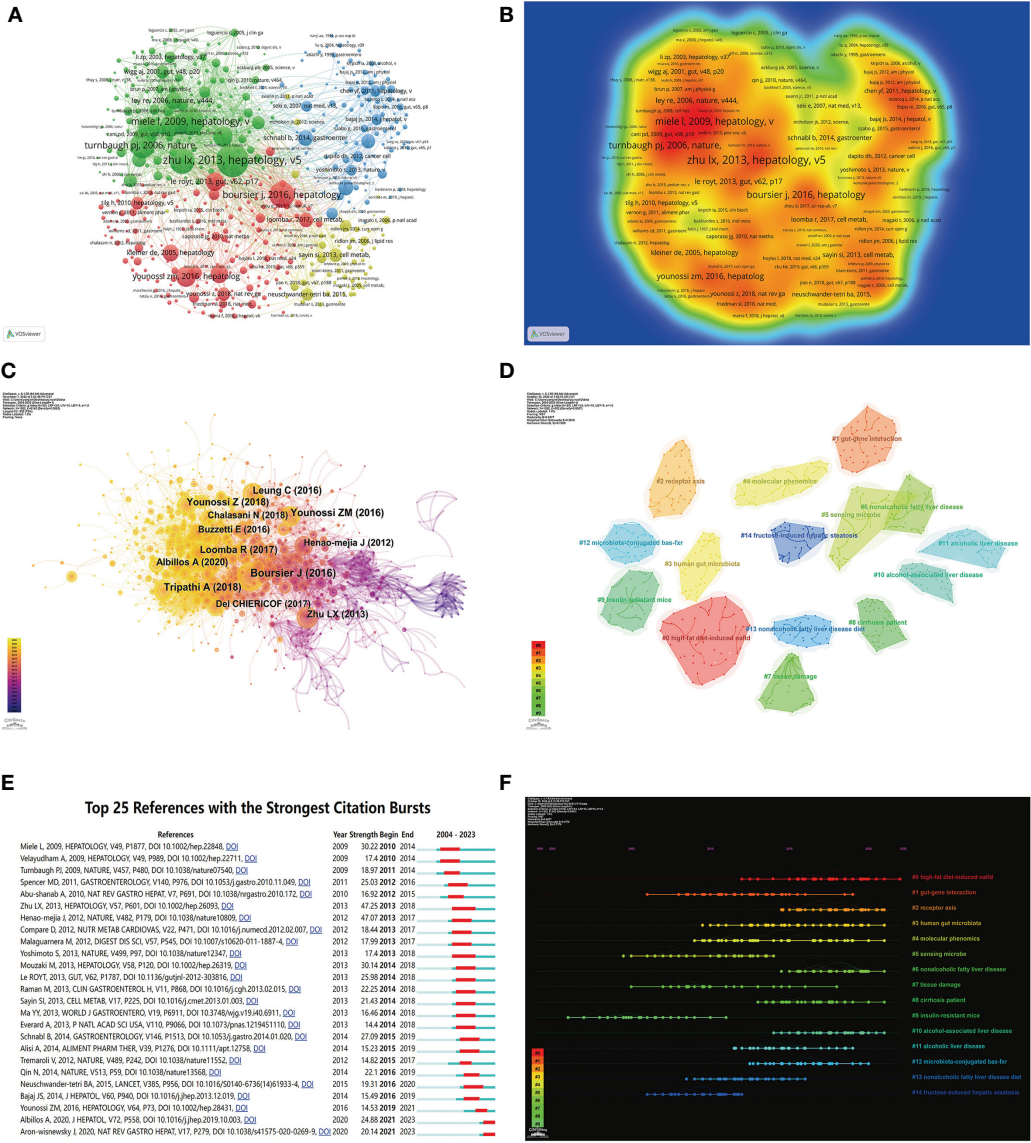
Visualization of co-cited literature on the gut-liver axis in NAFLD. **(A)** References co-citation network in the gut-liver axis in NAFLD based on VOSviewer. **(B)** The density visualization of co-cited references based on VOSviewer. **(C)** References co-citation network in the gut-liver axis in NAFLD based on CiteSpace. **(D)** Cluster Analysis of Co-cited References based on CiteSpace. **(E)** Top 25 references with the strongest citation bursts in the gut-liver axis in NAFLD. **(F)** A timeline of the 15 largest clusters in the gut-liver axis in NAFLD.

Co-cited references represent the degree of relationship between references. VOSviewer was used to find the top 5 references with the most co-citations (**Figures 7A, B**; **Table 6**). Among them, the most cited article was written by Lixin Zhu, pointing out that escherichia, which is related to endogenous ethanol production in the intestinal microbiota of NASH patients, was significantly increased, and the elevation of ethanol concentration was also observed in the blood of NASH patients, suggesting that the escherichia may be a risk factor promoting the progression of diseases from obesity to NASH.

**Table 6:** The top 5 most co-cited references related to the gut-liver axis in NAFLD.

Rank	First author	Journal	Year	Citations	TLS
1	Lixin Zhu (13)	Hepatology	2013	327	9723
2	Luca Miele (14)	Hepatology	2009	275	7738
3	Jérôme Boursier (15)	Hepatology	2016	256	7218
4	Jorge Henao-Mejia (16)	Nature	2012	256	7202
5	Peter J. Turnbaugh (17)	Nature	2006	249	6112

We used CiteSpace to further analyze the co-cited references, parameters were set as follows: time slicing (2004–2022), years per slice (1), node type (cited reference), selection criteria (k=25), and no pruning. As seen in As seen in **Figure 7C**, a co-occurrence network with a node number of 1,202, a connection number of 6,145, and a density of 0.0085 was obtained. We then performed a cluster analysis on the cited references based on the log-likelihood ratio (LLR) and 135 clusters were found. Only top 15 clusters are shown in the figure (**Figures 7D, F**). Among them, the clustering modularity Q=0.9477, and the average silhouette score S=0.5816, indicating this clustering is reasonable and the clustering structure is significant. The value of the cluster number represents the intensity of attention on the cluster topic within the discipline. The smaller the cluster value, the higher the attention. These clusters are mainly in 4 aspects. Firstly, animal models of NAFLD, including (#0 high-fat diet-induced NAFLD, #9 insulin-resistant mice, #13 nonalcoholic fatty liver disease diet, #14 fructose-induced hepatic steatosis). Secondly, the pathogenesis of NAFLD by the gut-liver axis, including (#1 gut-gene interaction, #2 receptor axis, #3 human gut microbiota, #12 microbiota-conjugated bas-fxr). Followed by examinations and tests commonly used in research in this field, including (#4 molecular phenomics, #5 sensing microbe, #7 tissue damage), and finally, the diseases related to NAFLD that have been studied in this field, including (#6 nonalcoholic fatty liver disease, #8 cirrhosis patient, #10 alcohol-associated liver disease, #11 alcoholic liver disease).

Burst detection of cited references represents a shift in research focus in a field. In CiteSpace, we set the parameters minimum Duration=2, γ=1, and screened 25 references with the strongest citation bursts, indicating their importance in the gut-liver axis in NAFLD-related fields (**Figure 7E**). Among those, the strongest citation burst was for the 2009 article *Increased intestinal permeability and tight junction alterations in nonalcoholic fatty liver disease*, this article proved for the first time that human NAFLD was associated with increased intestinal permeability, and this abnormality was associated with the increased prevalence of small intestinal bacterial overgrowth (SIBO) of patients, which is of great significance for studies of the gut-liver axis in NAFLD.

### 3.7 Keyword analysis

**Figure 8:**
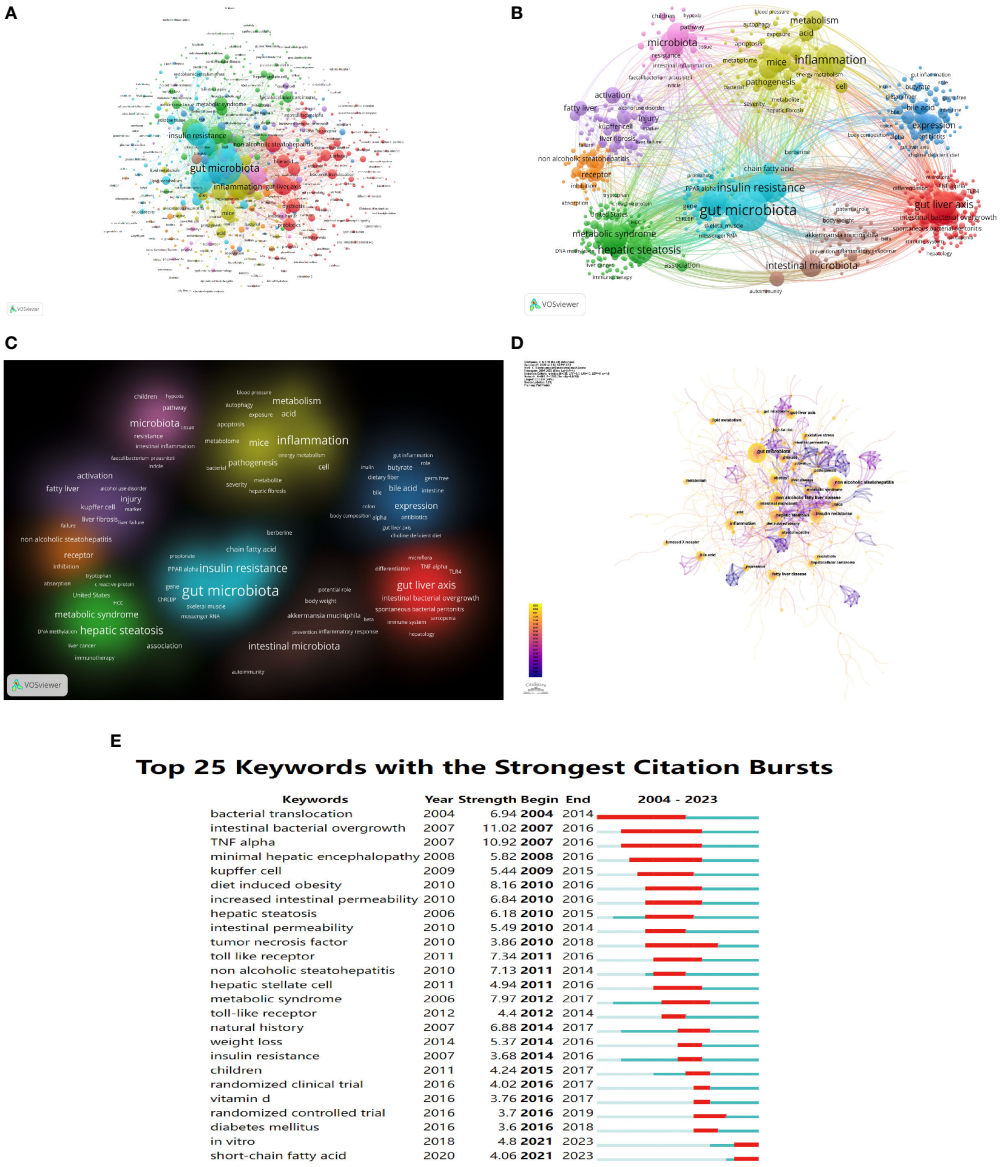
The mapping on keywords of the gut-liver axis research in NAFLD. **(A)** Network map of 701 keywords with frequency more than 5. **(B)** The cluster of keywords in the studies of the gut-liver axis in NAFLD (divided into 9 clusters by different colors.) **(C)** Density visualization of keyword clustering. **(D)** Visualization of keywords based on CiteSpace. **(E)** Top 25 keywords with the strongest citation bursts.

Keyword co-occurrence network could help us identify research hotspots and trends in a field. In this study, we used VOSviewer software to perform keyword analysis, the minimum number of occurrences of a keyword was set as 5, and a total of 701 keywords were extracted (**Figure 8A**, **Table 7**). 9 clusters were obtained from further cluster analysis of the keywords (**Figures 8B, C**), representing 9 research directions and study areas. The largest cluster is the cluster 1 (red), with 146 keywords, including the gut-liver axis, bile acid, dysbiosis, probiotics, bacterial translocation, intestinal permeability, LPS, Toll like receptor, TNF-α, etc. Followed by the cluster 2 (green), with 123 keywords, including non-alcoholic fatty liver disease, hepatic steatosis, hepatocellular carcinoma (HCC), liver cancer, diabetes mellitus, metabolic syndrome, cardiovascular disease (CVD), etc. Cluster 3 (blue) has 86 keywords, mainly including expression, NF Kappa b, FXR, bile acid metabolism, nuclear receptor, obeticholic acid, vitamin d receptor. Cluster 4 (yellow) has 83 keywords, mainly including Pathogenesis, inflammation, mechanism, diet, glucose, acid, metabolism, bacterial, gut microbiome, apoptosis, autophagy. Cluster 5 (purple) has 76 keywords, mainly including activation, fatty liver, injury, kupper cell, liver fibrosis, liver failure. Cluster 6 (light blue) has 74 keywords, including gut microbiota, obesity, insulin resistance, lipid metabolism, oxidative stress, chain fatty acid, and adipose tissue. Cluster 1 mainly reflects the definition of the gut-liver axis, and Cluster 3, 4, 5 mainly reflects the pathogenesis of the gut-liver axis in NAFLD and some drug targets under development. The keywords in Cluster 2 and 6 mainly reflect the diseases related to the gut-liver axis in NAFLD and the relationship between gut microbes and metabolic diseases.

**Table 7:** Top 10 keywords in terms of frequency in the gut-liver axis in NAFLD.

Rank	Keywords	Occurrences	TLS
1	gut microbiota	774	7172
2	inflammation	422	4063
3	insulin resistance	364	3562
4	obesity	349	3224
5	hepatic steatosis	325	3180
6	gut-liver axis	270	2597
7	microbiota	264	2536
8	nonalcoholic fatty liver disease	260	2553
9	fatty liver disease	236	2179
10	nonalcoholic steatohepatitis	225	2192

We also use CiteSpace to visualize the keywords co-occurrence network, a map with 661 nodes, 1,383 connections, and 0.063 density was obtained (**Figure 8D**). Burst detection analysis of the keywords found that studies on short-chain fatty acid, *in vitro*, randomized controlled trial in clinical, and diabetes mellitus are the latest keywords (**Figure 8E**), indicating these areas are research hotspots in recent years, and it also suggests future research focuses.

## 4 Discussion

In this study, 1,891 literature related to the NAFLD the gut-liver axis retrieved from the Web of Science core database was analyzed with VOSviewer, CiteSpace and Excel. The number of papers published in this field generally showed an upward trend in the past 20 years, and the average annual co-citations were increasing year by year. Before 2009, there were few studies in this field. After 2009, researchers gradually started to pay attention to the role of the liver gut axis in NAFLD. Especially after 2018, this field has entered a relatively mature stage of development.

China, the United States, and Italy are currently main countries doing research related to the gut-liver axis in NAFLD. In the past 20 years, China has published the most articles, while its average citation was only 17.09, suggesting that Chinese researchers should devote more energy to work on high quality articles. The number of publications of the United States is also much higher than that of other countries, and it has advantages in terms of number of papers cited, H-index and TLS, indicating that the United States is more prominent in research of the field. Centrality represents the importance of the node in the network. Among the top 10 institutions in the centrality ranking, 7 institutions are from the United States, two are from France and one is belonged to Belgium, which further explains the leading position of the United States in this research field. The connecting links between nodes represent the intensity of cooperation between countries, and close cooperation between countries is conducive to the further development of the research.

Among the top 10 authors with the largest number of publications, Dr. Bernd Schnabl ranked first, followed by Jasmohan S. Bajaj and Ki Tae Suk. Dr. Bernd Schnabl reviewed the connection between the gut-liver axis and liver diseases and pointed out that we could determine the type of liver disease and its possible progression based on microbial changes, provided a new idea for the diagnosis and prognosis of liver-related diseases (18). His recently published article studied the commensal fungi in the gut-liver axis and suggested that histological disease severity in patients with NAFLD is associated with changes in the fecal mycobiome, indicating us intestinal fungi could be an attractive target to attenuate NASH (19). Patrice D. Cani is the most co-cited researcher, he is an expert in the field of gut microbiota and his academic interest is mainly focusing on the interaction between gut microbes and the host in obesity, type 2 diabetes, cardiovascular disease, and metabolic diseases. He found that metabolic LPS can trigger obesity and insulin resistance, which are the most common cause of NAFLD. Therefore Prof. Patrice D. Cani begun to pay attention to the research of NAFLD in recent years. Jasmohan S. Bajaj also has a good record in the number of publications and co-citations, who has made important contributions in the field of the gut-liver axis in NAFLD. Jasmohan S. Bajaj (20) studied the changes in the gut microbiome in obese and non-obese NAFLD patients, and found that in non-obese NAFLD patients, the changes of intestinal microbes and metabolites were related to the degree of liver fibrosis, and then confirmed this finding with three animal models, suggesting that changes in gut microbes and metabolites in non-obese NAFLD patients can be used as markers and therapeutic targets of fibrosis. Among the top 10 co-cited authors, Fredrik Bäckhed, Lixin Zhu, and Peter J. Turnbaugh all have centrality value greater than 0.1, indicating that these three authors have great influence in the field.

Keywords reflect the core theme of the article, rank articles according to the frequency of co-occurrence could facilitate the analysis of the research focus in the field (21). In this study, we can see that the research focus in this field is mainly on the gut microbiota, inflammation, insulin resistance, etc. The interaction between the gut microbiota and the liver is called the gut-liver axis, it is not surprising then that the frequency of gut microbiota is so high in this study. Changes of intestinal flora will cause increase in intestinal permeability, and promote the binding of bacteria and their metabolites to TLRs receptors of the liver, thus induce pro-inflammatory factors such as TNF-α and IL1-β to activate inflammatory responses (22, 23). Inflammation is an important pathological manifestation in the progression of NASH in NAFLD patients, and it may even exacerbate the deterioration of NASH into HCC. Another clinical study also found that the increase of LPS in NAFLD patients would further aggravate inflammation and lead to insulin resistance (24). Insulin resistance is also an important pathological manifestation of NASH patients, and it also interacts with the gut-liver axis. Studies (25) have found that in the intestinal tract of mice with insulin resistance, the expression of tight junction proteins decreased, and the content of intestinal bacterial LPS increased.

The burst detection analysis of keywords can represent the research frontiers in the field within a certain period, which could help researchers understand the dynamic changes of the frontiers of disciplines (26). Burst detection analysis shows that short-chain fatty acid (SCFA) was a research hotspot in the past two years. SCFAs, including acetate, butyrate, and propionate, are metabolic products of intestinal bacteria. They are mainly produced in the distal colon and serve as substrates for gluconeogenesis and lipogenesis, providing nutrients and energy to the host (27). SCFAs stimulate the secretion of peptide YY (PYY) and GLP-1 by activating G-protein coupled receptors (GPRs) GPR41 and GPR43, thereby suppressing appetite and reducing energy intake (28). A clinical trial of obese people found that acute supplementation with inulin-propionate ester significantly increased postprandial serum PYY and GLP-1 and reduced energy intake, and long-term supplementation could reduce body weight and increase lipid content in hepatocytes (29). Similarly, long-term butyrate consumption can prevent HFD-induced hepatic steatosis and insulin resistance in mice, by reducing their food intake. Meanwhile, SCFAs can improve HFD-induced NAFLD by increasing fatty acid oxidation of brown adipose tissue (BAT) *via* the brain-gut axis (30). In addition to serving as substrates for energy production, SCFAs play an important role in maintaining intestinal homeostasis. Evidences showed that in both adults and animals, NAFLD is associated with an increased ratio of *Firmicutes/Bacteroidetes* (31–33) and increasing the intake of SCFAs through a high-fiber diet could promote the growth of the Bacteroidetes and maintain the integrity of the intestinal barrier by upregulating the expression of tight junction proteins (34). In addition, SCFAs may act as signaling factors to activate AMP-activated kinase (AMPK) to promote TG hydrolysis and β-oxidation of fatty acids, thereby reducing hepatic lipid deposition and liver inflammation in mice (35). Therefore, supplementing SCFA may be a promising strategy to prevent or treat NAFLD (36, 37). Clinical trial was another hot topic detected in burst detection analysis. Among the current clinical trials, the most popular intervention is lifestyle intervention, including shifts in dietary patterns (38–40) and exercise (41, 42), while intrahepatic fat loss, changes in serum biomarkers or gut microbiota are the most common primary outcomes. Additional supplemental dietary fiber (43, 44), fecal microbiota transplantation (45), probiotics (46), and some antibiotics (47, 48), have also received considerable attention. Several drugs that have proven effective in animal studies in regulating the liver-gut axis, have also shown clinical potential for the treatment of NAFLD (49). A phase II a trail (50) showed that Lubiprostone improved AST and hepatic steatosis in patients with NAFLD and constipation by decreasing intestinal permeability. Another randomized controlled trial in NAFLD patients (51) showed that oligonol, a lychee extract that has been shown in mice to alleviate NAFLD by modulating the gut microbiota, could increase the abundance of SCFAs in the gut and reduced hepatic steatosis in patients. Besides, several studies have also focused on gut microbiota-related effects of drugs that may be effective in treating NAFLD, such as Aldafermin (an analog of the gut hormone FGF19) (52, 53) and the FXR agonist PX104 (54).

Cluster analysis of keywords and co-cited references can help researchers better understand the research status in this field (55). According to the clustering results, the research status of the gut-liver axis in NAFLD could be summarized as follows:

### 4.1 Animal models of NAFLD

Animal model is an important tool in medical research, and choosing an appropriate animal model is the key to NAFLD drug development. Currently, the commonly used NAFLD animal models include: **a.** Diet-induced animal models, including nutrient deficient models such as methionine- and choline-deficient (MCD) diet, choline-deficient L-amino acid defined (CDAA) diet, and high-fat diet (HFD)-induced models such as Western-diet mice, fructose-induced mice and HFD induced model; **b.** Chemical Models, such as Streptozotocin(STZ) -HFD induced NAFLD model, Carbon Tetrachloride(CCL4)-induced model and Diethyl nitrosamine(DEN) model; **c.** Genetic models, such as combine Type 2 Diabetes Mellitus models(ob/ob mice or db/db mice) or Atherosclerosis models with HFD diets. No matter which modeling it is, the need for dietary induction is impossible to be avoided. However, the type of diet could also affect the changes in the intestinal microbial flora (56). Therefore, when studying the influence of the gut liver axis in NAFLD, it is also necessary to carefully select an appropriate animal model. According to current researches, the animal model of NAFLD still cannot completely replicate the onset of the disease in clinical patients, and the results of animal experiments need to be further verified by clinical trials such as randomized controlled trials.

### 4.2 The pathogenesis of the gut-liver axis in NAFLD

The pathogenesis of NAFLD is complex and it is the result of multiple factors including diet, metabolic factors, gut microbial flora, and genetic factors (57). GVB is an important cause of disease progression in NAFLD patients (58), mainly manifested as the imbalance of intestinal flora, the increase of intestinal permeability, the translocation of bacteria and their products to the liver, thus inducing the activation of Kupper cell to release inflammatory factors (59), which triggers the inflammatory response. Multiple studies have found that Escherichia (14) and Klebsiella pneumoniae (60) in the intestines of NAFLD patients were significantly increased, which would produce high-concentrations endogenous alcohol, further accelerating intestinal barrier damage and fat accumulation in the liver. Bile Acids (BAs) are synthesized from hepatic cholesterol and released into the small intestine in the form of bile salts (61), it could prevent bacterial overgrowth while maintain microbial homeostasis in the intestine. At the same time, BAs can act as ligands for FXR and G protein-coupled bile acid receptor 1 (TGR5), regulating lipid, glucose, and energy metabolism (62), it is also involved in many cascade reactions on the gut-liver axis.

Targeting the gut-liver axis has become an emerging strategy for the prevention and treatment of NAFLD. Several potential treatments for NAFLD are currently being developed, including: a. Antibiotics. Antibiotics are controversial drugs for NAFLD. Early-phase clinical studies (47, 63) have shown that short-term use of rifaximin (a rifamycin antibiotic) can improve ALT levels in patients with NAFLD. While several animal studies (64, 65) have shown the efficacy of antibiotics to treat NAFLD, caution should be exercised in the utilization of these antibiotics as deleterious effects on beneficial bacteria species and the appearance of antibiotic-resistant strains (5). Therefore, further therapeutic strategies for optimizing antibiotic treatment are needed. b. Probiotics, Prebiotics and Symbiotics. Several interventions have addressed the overgrowth of harmful bacteria by promoting the growth of beneficial bacteria. As early as in 2009, Pr. Patrice D. Cani, the author with the most citations in this study, found that administration of oligofructose prebiotics could reduce intestinal permeability and improve liver inflammation and oxidative stress levels in ob/ob mice (66). However, most of these efficacies are proved in animal experiments, and more clinical trials are required for verification. c. Targeted bile acid metabolism. Currently FXR is the most studied transcriptional factor in bile acid metabolism, activation of FXR could induce metabolic effects and reduce steatosis and inflammation. Obeticholic acid, a first-in-class FXR agonist, has been proven to modify the liver pathological progression (steatosis, inflammation and hepatocyte ballooning, fibrosis) of NASH animal models (7, 67, 68). However, in the phase 3 randomized placebo-controlled trial (69), only 23% of participants present with the improvement in liver fibrosis after administration for 18 months, and approximately half of those participants treated with 25 mg OCA developed pruritus (70). d. Others: TLR-4 antagonists, FGF-19 agonists, and GLP-1 agonists, etc.

### 4.3 NAFLD-related diseases

NAFLD is a multisystem disease that affects a variety of extra-hepatic organs, and causes dysregulation of multiple biological pathways (71). NAFLD-related diseases mainly include liver complications and metabolic diseases, such as type 2 diabetes mellitus (T2DM), CVD, and chronic kidney disease (CKD). As time progresses, the end-stage of NAFLD may lead to some liver complications, such as cirrhosis and HCC. Clinical studies (72) have shown that the common features of patients of NAFLD with HCC are the lack of protective bacteria in the stool and the aggravation of intestinal inflammation. In addition, the increase of secondary bile acids produced by the microbiota will further aggravate liver inflammation and damage, and accelerate the deterioration from NASH to HCC (73). Therefore, improving intestinal dysbiosis and regulating BA metabolism through the intervention of the gut liver axis may be an effective method to prevent the progression of HCC.

Evidence (74) shows that NAFLD is an important risk factor for various metabolic-related extrahepatic diseases. NAFLD and T2DM share common pathogenic mechanisms such as lipotoxins, mitochondrial function, cytokines and adipocytokines, bile acid metabolism (75). Therefore, these two diseases commonly occur together, and drugs for the treatment of T2DM such as thiazolidinediones (glitazones), SGTL2i and GLP-1RA are also commonly used for NAFLD (28, 76). The leading cause of mortality among patients in NAFLD is CVD, and low-grade inflammation, gut microbial imbalance and oxidative stress may be the main mechanism of NAFLD-caused CVD. Trimethylamine-N-oxide (TMAO) is an intrahepatic metabolite of choline by the gut microbiome, and it is a significant marker of atherosclerosis and increased risk of CVD (77). Meanwhile, its circulating concentration is also considered related to the severity of NAFLD (78). Elevated systemic TMAO levels may also have adverse effects on the kidneys (79), causing CKD through the crosstalk of gut-liver-kidney, and damaged kidneys will further aggravate the NAFLD by destroying the intestinal barrier and activating RAAS (80). In short, gut microbiota, microbiota-derived products, and epithelial barrier integrity represent common pathological mechanisms in NAFLD/NASH and its metabolic comorbidities. Therefore, targeting the gut liver axis is a promising therapeutic strategy for NAFLD-related diseases.

In general, although there is a large amount of animal experiment evidence indicating the importance of the gut-liver axis in NAFLD, there still lacks large-scale clinical data to prove the safe dosage and clinical efficacy of drugs targeting the liver-gut axis. This will also be the focus of future researchers.

## 5 Conclusion

According to this bibliometric analysis, research on the gut-liver axis in NAFLD started since 2004. With researchers’ in-depth understanding of gut microbes, new therapeutic targets in the gut-liver axis have a potentially wide application in treating NAFLD. Current research hotspots mainly include animal models of NAFLD, therapeutic targets and mechanism exploration on the gut-liver axis, consisting of gut microbial dysbiosis, impaired intestinal barrier and bile acid metabolism. At present, short-chain fatty acids and clinical research especially randomized controlled trials are new research hotspots in this field.

## Data availability statement

The original contributions presented in the study are included in the article/supplementary material. Further inquiries can be directed to the corresponding authors.

## Author contributions

SY and RY designed the study. SY, DY, YQ, and SG conducted the literature search. DY and JL analyzed the data and wrote the paper. SY and DY approved the final manuscript. All authors contributed to the article and approved the submitted version.
